# Supplementary figures and images for: Severe anaemia complicating HIV in Malawi; Multiple co-existing aetiologies are associated with high mortality
Source: PLoS One. 2020 Feb 25;15(2):e0218695. doi: 10.1371/journal.pone.0218695 (PMC7041863; doi:10.1371/journal.pone.0218695)

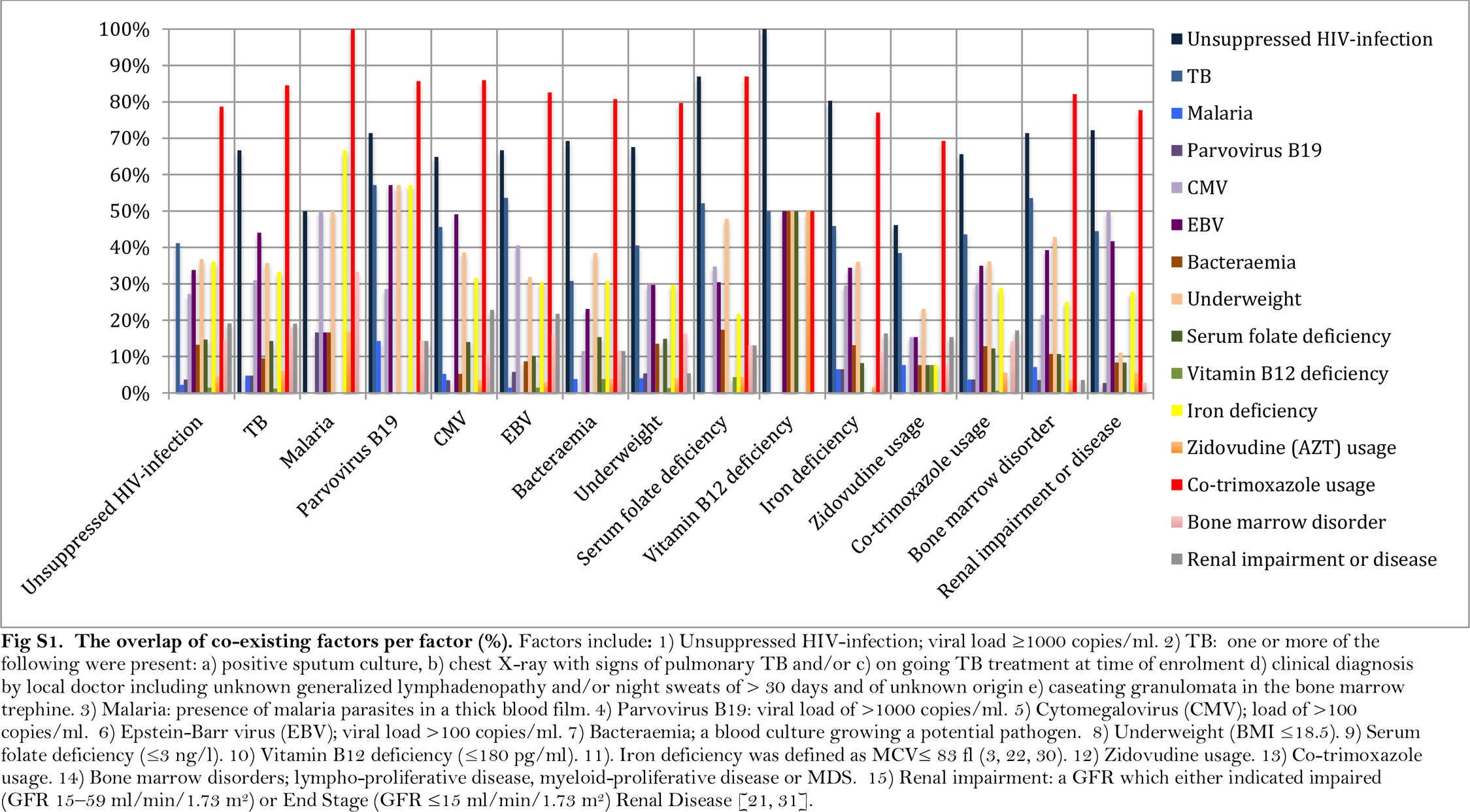

Supplement: S1 Fig — (TIF) [file pone.0218695.s002.tif]
