# Supplementary material for: Severe anaemia complicating HIV in Malawi; Multiple co-existing aetiologies are associated with high mortality
Source: PLoS One. 2020 Feb 25;15(2):e0218695. doi: 10.1371/journal.pone.0218695 (PMC7041863; doi:10.1371/journal.pone.0218695)
Supplement: S1 Table — (DOCX) [file pone.0218695.s003.docx]

**S1 Table. Distribution of co-existing factors between mortality within 60 days and overall mortality in HIV-infected adults in Malawi.**

|  | **Mortality ≤60 days** | **Mortality > 60 days** | **P-value** |
| --- | --- | --- | --- |
|  | **N= 81/101 (80%)** | **N= 20 (20%)** |  |
| Sex (female) | 48/81 (59.3% | 15/20 (75.0%) | 0.193 |
| Very severe anaemia (Hb < 50 g/l) | 35/81 (43.2%) | 5/20 (25.0%) | 0.136 |
| Hb (g/dl) (mean/SD) | 5.2 (1.3) | 5.2 (1.1) | 0.983 |
| **HIV** | | |  |
| CD4 ≤200 cells/mm^3^  (median/IQR) | 121.6 (32-370) | 90( 21-211) | 0.419 |
| Unsuppressed HIV-infection | 54/73 (74.0%) | 13/18 (72.2%) | 0.880 |
| On ART at enrollment | 38/81 (46.9%) | 8/20 (40.0%) | 0.578 |
| **Infection** | | |  |
| Malaria | 3/60 (0.05%) | 0/14 | 0.528 |
| Tuberculosis | 1/81 (1.2%) | 1/20 (5.0%) | 0.358 |
| Bacteraemia^1^ | 9/81 (11.1%) | 3/20 (15.0%) | 0.630 |
| Parvovirus B19 | 4/68 (5.9%) | 1/16 (6.3%) | 0.662 |
| Cytomegalovirus (CMV) | 23/68 (33.8%) | 7/16 (43.8%) | 0.456 |
| Epstein-Barr virus (EBV) | 34/68 (50.0%) | 2/16 (12.5%) | 0.006* |
| EBV/CMV co-infection | 13/68 (19.1%) | 2/16 (12.5%) | 0.534 |
| **Malnutrition** | | |  |
| Underweight | 33/68 (48.5%) | 8/13 (61.5%) | 0.390 |
| MCV ≤ 83 fl | 21/71 (29.6%) | 6/17 (35.3%) | 0.646 |
| Folate deficiency | 14/80 (17.5%) | 2/19 (10.5%) | 0.458 |
| **Medication** | | |  |
| Cotrimoxazole | 65/81 (80.2%) | 16/20 (80.0%) | 0.980 |
| Zidovudine | 5/38 (13.2%) | 1/9 (11.1%) | 0.869 |
| **Renal function** | | |  |
| Impaired (eGFR 15-60) | 8/72 (11.1%) | 0/19 | 0.305 |
| End stage (eGFR ≤15) | 8/72 (11.1%) | 2/19 (10.5%) | 0.305 |
| **Bone marrow** | | |  |
| Bone marrow disease | 13/32 (40.6%) | 2/4 (50.0%) | 0.720 |
| **Aetiology** | | |  |
| Co-existing aetiologies per patient (mean, SD) | 3 (1.0) | 3 (1.0) |  |

^1^ In the total cohort 28-blood cultures were positive, the most common organisms were E. coli (42.9%; 12/28) and non-Typhoid Salmonella (17.9 %; 5/28). Aetiologies for severe anaemia include: 1) Unsuppressed HIV-infection; viral load ≥1000 copies/ml. 2) TB: one or more of the following were present: a) positive sputum culture, b) chest X-ray with signs of pulmonary TB and/or c) on going TB treatment at time of enrolment d) clinical diagnosis by local doctor including unknown generalized lymphadenopathy and/or night sweats of > 30 days and of unknown origin e) caseating granulomata in the bone marrow trephine. 3) Malaria: presence of malaria parasites in a thick blood film. 4) Parvovirus B19: viral load of >1000 copies/ml. 5) Cytomegalovirus (CMV); load of >100 copies/ml. 6) Epstein-Barr virus (EBV); viral load >100 copies/ml. 7) Bacteraemia; a blood culture growing a potential pathogen. 8) Underweight (BMI ≤18.5). 9) Serum folate deficiency (≤3 ng/l). 10) Vitamin B12 deficiency (≤180 pg/ml). 11). Iron deficiency defined by MCV ≤ 83 fl. 12) Zidovudine usage. 13) Cotrimoxazole usage. 14) Bone marrow disorders; lympho-proliferative disease, myeloid-proliferative disease or MDS. 15) Renal impairment: a GFR which either indicated impaired (GFR 15–59 ml/min/1.73 m^2^) or End Stage (GFR ≤15 ml/min/1.73 m^2^) Renal Disease [22, 35] .
