# Supplementary material for: Severe anaemia complicating HIV in Malawi; Multiple co-existing aetiologies are associated with high mortality
Source: PLoS One. 2020 Feb 25;15(2):e0218695. doi: 10.1371/journal.pone.0218695 (PMC7041863; doi:10.1371/journal.pone.0218695)
Supplement: S2 Table — (DOCX) [file pone.0218695.s004.docx]

**S2 Table. Risk factors for 60-day mortality in HIV-infected patients with severe anaemia.**

|  | **Univariate** |  | **Multivariate** |  |
| --- | --- | --- | --- | --- |
|  | **HR** | **95%CI** | **HR** | **95%CI** |
| Very severe anaemia (Hb≤ 50g/l) | 1 | 0.7-1.6 |  |  |
| Gender (female) | 0.7 | 0.5-1.1 |  |  |
| **HIV** | | | | |
| Unsuppressed HIV-infection | 0.9 | 0.5-1.6 |  |  |
| **Infection** | | | | |
| Malaria | 1.4 | 0.4-4.5 |  |  |
| Tuberculosis | 2.1 | 0.02-1.5 |  |  |
| Bacteremia | 0.7 | 0.4-1.5 |  |  |
| Parvovirus B19 | 2.1 | 0.7-5.8 |  |  |
| Cytomegalovirus (CMV) | 1 | 0.6-1.6 |  |  |
| Epstein-Barr virus (EBV) | 2.1 | 0.7-5.8 | 1.6 | 1.0-2.7 |
| EBV/CMV co-infection | 1.3 | 0.7-2.3 |  |  |
| **Malnutrition** |  | | | |
| Underweight | 0.9 | 0.6-1.4 |  |  |
| MCV ≤ 83 fl | 0.8 | 0.5-1.3 |  |  |
| Folate defiency | 1.9 | 1.1-3.4 | 1.9 | 1.0-3.8 |
| **Medication** |  | | | |
| Cotrimoxazole | 0.9 | 0.5-1.6 |  |  |
| Zidovudine | 0.9 | 0.3-2.3 |  |  |
| **Renal function** |  | | | |
| Impaired (GFR 15-60) | 0.9 | 0.4-1.9 | 1 | 0.4-2.2 |
| End stage (GFR ≤15) | 2.2 | 1-4.5 | 2.7 | 1.2-6.2 |
| **Bone marrow** |  | | | |
| Bone Marrow disease | 1,1 | 0.9-1.3 |  |  |

Univariate and multivariate Cox regression outcome (Hazard Ratios 95% CI). End stage renal disease (GFR ≤15); HR 2,7 5% CI 1,2-6,2, p-value 0.042 was associated with 60 day mortality. Aetiologies for severe anaemia include: 1) Unsuppressed HIV-infection; viral load ≥1000 copies/ml. 2) TB: one or more of the following were present: a) positive sputum culture, b) chest X-ray with signs of pulmonary TB and/or c) on going TB treatment at time of enrolment d) clinical diagnosis by local doctor including unknown generalized lymphadenopathy and/or night sweats of > 30 days and of unknown origin e) caseating granulomata in the bone marrow trephine. 3) Malaria: presence of malaria parasites in a thick blood film. 4) Parvovirus B19: viral load of >1000 copies/ml. 5) Cytomegalovirus (CMV); load of >100 copies/ml. 6) Epstein-Barr virus (EBV); viral load >100 copies/ml. 7) Bacteraemia; a blood culture growing a potential pathogen. 8) Underweight (BMI ≤18.5). 9) Serum folate deficiency (≤3 ng/l). 10) Vitamin B12 deficiency (≤180 pg/ml). 11). Iron deficiency defined by MCV ≤ 83 fl. 12) Zidovudine usage. 13) Cotrimoxazole usage. 14) Bone marrow disorders; lympho-proliferative disease, myeloid-proliferative disease or MDS. 15) Renal impairment: a GFR which either indicated impaired (GFR 15–59 ml/min/1.73 m^2^) or End Stage (GFR ≤15 ml/min/1.73 m^2^) Renal Disease [22, 35] .
